# Deep-Learning-Based Automatic Detection of Photovoltaic Cell Defects in Electroluminescence Images

**DOI:** 10.3390/s23010297

**Published:** 2022-12-27

**Authors:** Junjie Wang, Li Bi, Pengxiang Sun, Xiaogang Jiao, Xunde Ma, Xinyi Lei, Yongbin Luo

**Affiliations:** 1 College of Information Engineering, Ningxia University, Yinchuan 750021, China; 2 China Telecom Tianyi Cloud Technology Co., Ltd., Chengdu 610000, China; 3 The Party School of Sichuan Provincial Committee of C.P.C, Chengdu 610072, China

**Keywords:** electroluminescence images, deep learning, defect detection, feature fusion

## Abstract

Photovoltaic (PV) cell defect detection has become a prominent problem in the development of the PV industry; however, the entire industry lacks effective technical means. In this paper, we propose a deep-learning-based defect detection method for photovoltaic cells, which addresses two technical challenges: (1) to propose a method for data enhancement and category weight assignment, which effectively mitigates the impact of the problem of scant data and data imbalance on model performance; (2) to propose a feature fusion method based on ResNet152–Xception. A coordinate attention (CA) mechanism is incorporated into the feature map to enhance the feature extraction capability of the existing model. The proposed model was conducted on two global publicly available PV-defective electroluminescence (EL) image datasets, and using CNN, Vgg16, MobileNetV2, InceptionV3, DenseNet121, ResNet152, Xception and InceptionResNetV2 as comparative benchmarks, it was evaluated that several metrics were significantly improved. In addition, the accuracy reached 96.17% in the binary classification task of identifying the presence or absence of defects and 92.13% in the multiclassification task of identifying different defect types. The numerical experimental results show that the proposed deep-learning-based defect detection method for PV cells can automatically perform efficient and accurate defect detection using EL images.

## 1. Introduction

In the past decade, solar PV energy, as a clean energy source, has gained considerable attention and has been developed greatly worldwide due to the increasing problems of environmental pollution and the energy crisis [1]. Currently, as a key renewable energy generation technology, PV power generation has achieved rapid development and has become a clean, low-carbon form of energy with great price competitiveness in many countries. The International Energy Agency (IEA) reports that from 2010 to 2021, global PV capacity increased from 17 GWdc to 172 GWdc, and by 2021, global PV installations increased by 19% annually, with the total cumulative installed PV capacity reaching at least 939 GWdc [2]. In particular, China’s solar energy consumption is expected to reach 33.0 Mtoe by 2023 [3]. However, the optimization, improvement and operating costs of PV power plants limit the long-term healthy development of the entire PV industry, of which PV cell defect detection has become a prominent problem.

**Figure 1:**
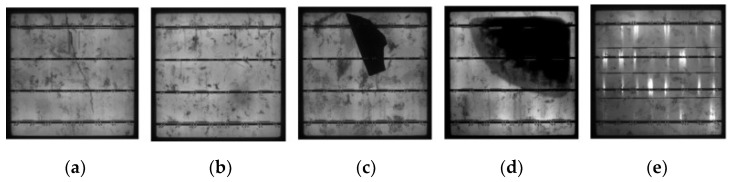
Different forms of defects in photovoltaic cells: (**a**) crack; (**b**) thick line; (**c**) fragment; (**d**) black core; (**e**) horizontal dislocation.

In fact, the production, processing and application of PV cell modules will produce various types of defects. As shown in Figure 1, contamination during the manufacturing process for PV cells can result in issues including dark cells, broken grids, fractures, lobes and chipped corners; in addition, PV cells may have hot spots and short circuits in operation [4]. Various defects in PV cells can lead to lower photovoltaic conversion efficiency and reduced service life and can even short circuit boards, which pose safety hazard risks [5]. As a result, PV cell defect detection research offers a crucial assurance for raising the caliber of PV products while lowering production costs.

To detect defects on the surface of PV cells, researchers have proposed methods such as electrical characterization [6], electroluminescence imaging [7,8,9], infrared (IR) imaging [10], etc. EL imaging is frequently utilized in solar cell surface detection studies because it is rapid, non-destructive, simpler and more practical to integrate into actual manufacturing processes [11]. EL imaging is mainly based on the electroluminescence principle of silicon materials for detection. By adding forward bias to a crystalline silicon cell module, the module will emit light of a certain wavelength, and a charge-coupled device image sensor can capture the light in this wavelength range and image it [12]. In order to improve the quality and efficiency of PV defect detection and promote the sustainable development of the PV industry and new energy applications, the use of cutting-edge computer technology to automatically perform the intelligent detection of defects is a necessary technical means. With the gradual deepening and improvement of image analysis technology and deep learning technology, the combination of computer vision technology and surface defect detection is becoming more and more closely applied.

In many fields, including computer vision and speech recognition [13], deep learning has achieved considerable success since it was first introduced by Hinton’s research team [14]. Deep learning methods far outperform traditional image processing algorithms in terms of accuracy and performance for tasks such as target detection and image recognition. In 2012, deep learning techniques made a large impact in machine vision, are widely used in various industrial scenarios and have become the mainstream methods for defect detection, e.g., AlexNet [15], VGG [16], GoogLeNet [17], ResNet [18], DenseNet [19], MobileNet [20], YOLO [21], etc. Defect detection methods based on deep learning technology with high accuracy and no damage can satisfy the needs of industrial application sites.

Deep learning methods have steadily been applied to industrial defect detection studies in recent years, and many scholars have studied the automatic detection of PV cell defects based on EL imaging methods. Deitsch et al. [22] proposed two deep-learning-based methods for the automatic detection of PV cell defects with convolutional neural networks (CNNs) and SVMs; the results showed that CNN classifier detection has higher accuracy. Rahman et al. [23] proposed CNN architecture and CNN integration; integrated learning not only improves accuracy but also reduces the risk of relying on a single model. However, when the CNN level is too deep, using BP propagation to modify the parameters will change the parameters near the input layer more slowly. Using the gradient descent algorithm will easily make the training results converge to the local minimum instead of the global minimum, and the pooling layer will lose a lot of valuable information and ignore the correlation between the local and the whole. A deep-learning-based defect diagnosis model was proposed, and a Hessian-matrix-based defect feature extraction method and a multi-scale line detector-based defect feature enhancement method were used to improve the performance, achieving a classification accuracy of 93% [24]. Akram et al. [25] proposed a new method for identifying EL image defects using an optical CNN structure, combining data enhancement and regularization strategies to expand the training dataset; the model achieved a classification accuracy of 93.02% and consumed less computational power and time. Huang et al. [26] proposed a multi-round PSOPruner to automatically search for the optimal DCNN pruning scheme, which deployed the PSO algorithm as a search engine while employing a multi-round trick to speed up and simplify the search process. Wang et al. [27] proposed a lightweight dual-stream defect detection network (DDDN) with a classification accuracy of 88.26%, accelerated using a field-programmable gate array (FPGA) based on a developed dual-stream parallel computing architecture (DPCA). Researchers have continued to improve on a single deep learning model to optimize the training process and reduce computational consumption time; however, model training relies on a large number of training datasets to ensure a balanced distribution of different types of data. In recent years, migration learning has shown satisfactory results with limited training datasets and on smaller, more practical datasets. Demirci et al. [28] proposed a defect detection model based on migration learning. It showed good results in processing EL images with simple backgrounds; however, the accuracy decreased significantly in complex backgrounds. Tang et al. [29] proposed an evolutionary algorithm which combines traditional image processing techniques, deep learning, migration learning and deep clustering; this fine-tuned model can detect new defects with high accuracy. Fan et al. [30] proposed a migration learning and ResNet-based microcrack detection method, which combines feature fusion and incorporates a self-attention mechanism to aggregate low-level features and deep semantic strong features to significantly improve defect detection.

In summary, deep learning techniques have achieved good results in the detection of defects in PV cells but can only basically satisfy the requirements of practical industrial scenarios; moreover, two difficulties in the existing research still need to be further addressed: (1) It is difficult to collect data from the actual application scenarios of PV cells, especially samples with defects, and there are flaws of small and unbalanced datasets; (2) traditional detection models and existing models based on the accuracy and efficiency of defect detection cannot fully achieve the practical requirements.

To solve the above problems, this paper proposes a deep-learning-based solution for the automatic defect detection of PV modules based on electroluminescent images, with the following key contributions.

(1) Adopting data augmentation and weight class to mitigate the effects of small data volume and data imbalance on the model performance, respectively.

(2) The fusion network based on ResNet152–Xception enhances the model’s feature extraction capability. Hybrid pooling is introduced to avoid the defects of traditional single pooling.

(3) Embedded CA to effectively improve the classification accuracy of the model.

(4) We experimented on two global public datasets to validate the model from the perspective of dichotomous and multiclassification tasks, respectively. In addition, to the best of our knowledge, this is the first time that the model has been applied to a dataset with no defects and nine defect types in studying the PV defect identification problem.

The rest of this paper is organized as follows. The models and methods used for the experiments are given in Section 2. The experimental steps and details are given in Section 3. The results obtained from the experiments are reported and discussed in Section 4. Finally, conclusions and future work are given in Section 5.

## 2. Methodology

Data are the basis of deep learning method research, and solving the small sample problem and imbalance problem is the most critical research point in PV cell defect recognition. Data enhancement techniques are widely used in the field of industrial defect surface detection; however, defect forms designed for a specific scene are difficult to generalize to other scenes and cannot fundamentally solve the problem. Therefore, the introduction of a migration learning model to achieve PV cell defect recognition enhances the feature extraction ability of the model to achieve a better classification effect on the one hand; on the other hand, the weights are directly loaded into the new model to reduce the cost of deep neural network training.

ResNet is optimized in terms of network structure, utilizing residual blocks to ensure good network performance even in deeper networks. In addition to the network depth problem, how to extend the neural network without increasing the computational cost is a key issue. Inception sets differently sized convolution kernels in each layer of the model and performs dimensionality reduction with 1 × 1 convolution. Xception improves on Inception by mapping spatial correlations for each output channel separately and then performs 1 × 1 convolution to obtain cross-channel correlations, separating the relationships on the channels from the spatial relationships for identification. Therefore, we chose to fuse the features of the two neural network models, ResNet and Xception, to combine the feature representations extracted from the deeper network level by ResNet and from the wider network level by Xception, enhancing the information extraction capability for yet defective regions.

**Figure 2:**
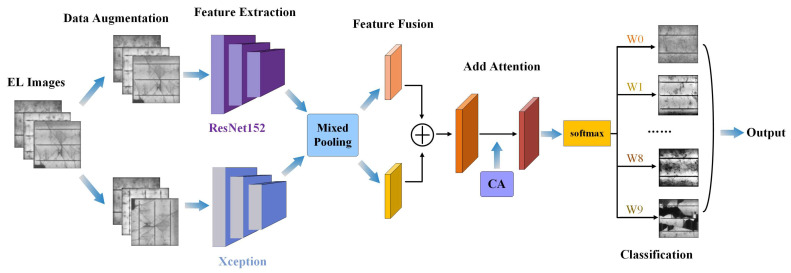
Overall architecture of the model.

Figure 2 shows the overall framework diagram of our experiment design. First, the images input to the training set were augmented with data enhancement method; second, the preprocessed data were input to ResNet152 and Xception pre-training models to extract features, and the features are pooled using hybrid pooling method; then the hybrid pooled features were combined along the spatial dimension to complete the feature fusion; subsequently, the features are fused with CA, and the location information is embedded into the channel attention; finally, Classweight is introduced in the classification layer to weight the classification probabilities of different categories, and the maximum probability is selected to input the classification prediction results.

### 2.1. Transfer Learning

Transfer learning [31] exploits the correlation between data to transfer the knowledge learned by the model on the source domain, *Ds*, and source task, *Ts*, to the target domain, *Dt*, and target task, *Tt*, and to enhance the prediction of the target task learning function *ft(·)* with the knowledge of *Ds* and *Ts* in the case that *Ds ≠ Dt* or *Ts ≠ Tt*. The domain consists of the feature space *X* and the probability distribution *P(X)*, which generates these data, and the task consists of the labeling space *Y* and the learning function *f(·).*

**Figure 3:**
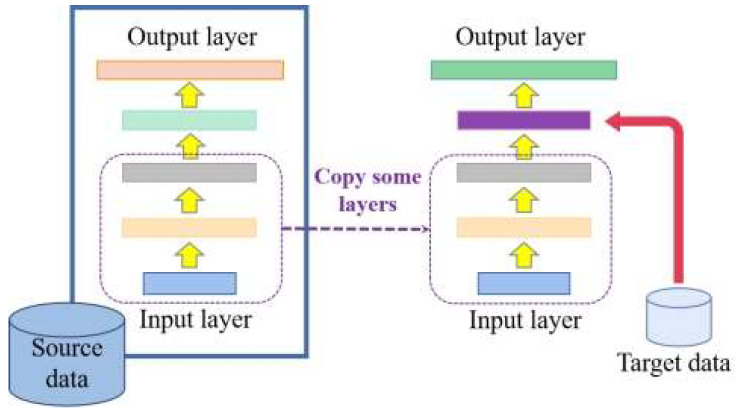
Transfer learning based on the model.

In this paper, we introduce a model-based learning approach in migration learning, where the parameters of the already trained model are migrated to the new model to help with training. The first n layers of the model are frozen and are not involved in training, and only the last few layers of parameters are trained after copying the weights, thus reducing the training time cost. The model-based migration learning model is shown in Figure 3.

### 2.2. ResNet152 Model

**Figure 4:**
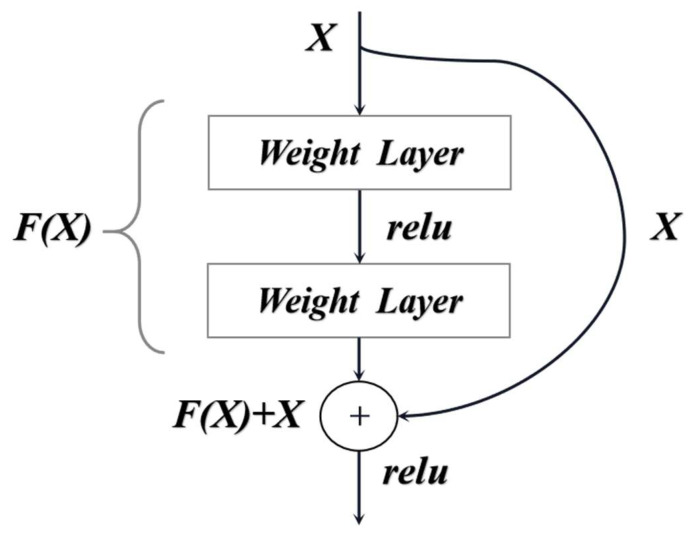
Structure of residual unit.

Deep residual networks (such as ResNet) solve the degradation problem caused by increasing the number of network layers in neural networks by stacking residual structures and effectively using the information of multiple layers in the network [18]. ResNet forms a residual unit by partially convolving layers and a short connection and then by stacking the residual units to form a residual network, which only needs to learn the residual function during the training process without adding additional parameters and computational complexity. The residual unit is shown in Figure 4, where *X* denotes the input of the residual block; *F(X)* denotes the mapping result of *X* after two layers of weights; relu is the modified linear unit; and the output of the residual block is *F(X)* + *X*.

The ResNet152 model used in this study was a ResNet network with 152 layers, which is 8 times deeper than VGG-19, but with a lower complexity and better ability to extract features [32]. The ResNet152 network uses a three-layer convolutional structure to form one residual network cell, with four feature extraction layers, Conv2_x, Conv3_x, Conv4_x and Conv5_x, corresponding to 3, 8, 36 and 3 residual network cells, respectively, plus the input convolutional layer Conv1 and the fully connected convolutional layer FC, for a total of 152 convolutional layers.

### 2.3. Xception Model

Google has proposed an improved Xception (Extreme Inception) to Inception v3, the ultimate Inception [33]. Depthwise separable convolution is used to replace the convolution operation in the original Inception v3. The problem of incomplete separation of channel correlation and spatial correlation is effectively solved, and the model is improved while maintaining the same number of parameters as InceptionV3.

The Xception network consists of 14 modules, including 36 convolutional layers, of which except for the first 2 convolutional layers and the convolutional layers connected by residuals, all adopt depth-separable convolution, and the basic network is constructed by stacking depth-separable convolution. The introduction of depth-separable convolution can effectively reduce the number of parameters to reduce the complexity of operations; moreover, the linear residual connection is used in all modules except the first and the last module, which can effectively solve the degradation problem caused by the network being too deep. The output of GlobalAveragePooling in the last module, i.e., a one-dimensional vector of length 2048, was used in this study.

**Figure 5:**
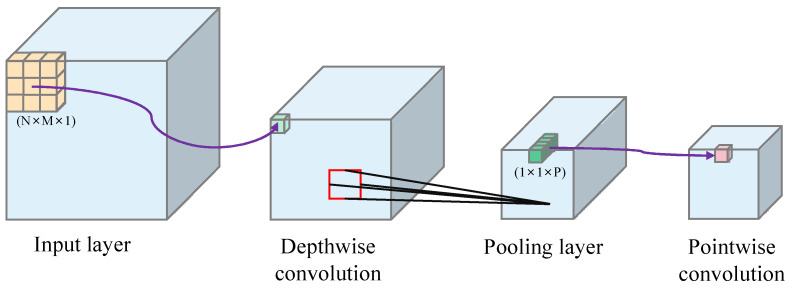
Structure of depthwise separable convolution.

The structure is shown in Figure 5. (1) Depthwise convolution convolves each input feature channel individually, assuming that the number of input feature maps is S and the convolution kernel size is k × k so that each input feature map will correspond to a separate k × k convolution kernel for convolution and output S feature maps; (2) pointwise convolution uses a standard convolution of 1 × 1 to correlate the feature channels between correlation output features.

### 2.4. CA

In image feature extraction, attention mechanisms can enhance feature selection. Position-based attention mechanisms act in two ways: one is by large-scale kernel convolution, such as squeeze excitation (SE) and the convolutional block attention module (CBAM); two is to decompose feature images, such as CA.

**Figure 6:**
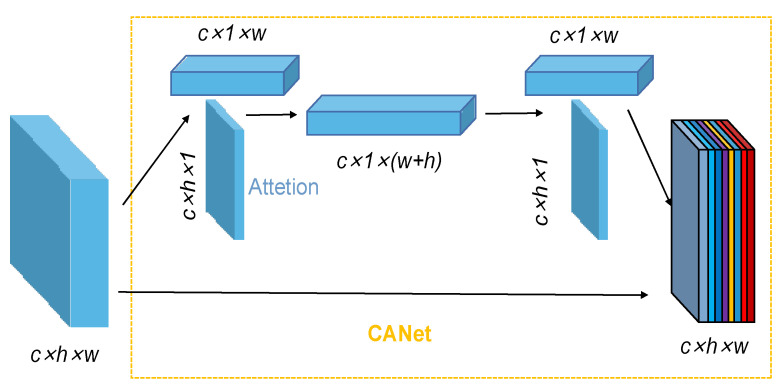
CA module.

Compared with the large-scale kernel convolution operation to obtain spatial information, decomposing the feature image can make full use of the captured location information so that the region of interest and the relationship between channels can be accurately and effectively captured. The overall flow of the CA mechanism module is shown in Figure 6, which decomposes the feature image into two one-dimensional codes through a two-dimensional global pool operation to effectively capture the location information and channel relationship; the specific location of the target is analyzed, and the related feature values are output.

The CA structure obtains accurate location information by two-dimensional encoding, which includes two steps of coordinate information embedding and coordinate attention generation, and can effectively enhance the performance of the deep network. The CA mechanism is processed by first encoding each channel along the horizontal and vertical directions; the calculation process is as follows:

(1) $$z_{c}=\frac{1}{H\times W}\sum_{i=1}^{H}\sum_{j=1}^{W}x_{c}(i,j)$$

In Equation (1), *H* is the height of the feature map, and *W* is its width; $x_{c}$ is the feature map of the *c*th channel. A pair of direction-aware feature maps of *c* × 1 × *w* and *c* × 1 × *h* in size is obtained as:

(2) $$z_{{}^{c}}^{h}=\frac{1}{W}\sum_{i=0}^{W}x_{c}(h,i)\;;\;z_{{}^{c}}^{w}=\frac{1}{H}\sum_{j=0}^{H}x_{c}(j,w)$$

In Equation (2), $z_{{}^{c}}^{h}$ is the output of the *c*th channel with height *h*; $z_{{}^{c}}^{w}$ is the output of the *c*th channel with width *w*. The global perceptual field is obtained after the above transformation, and precise position information can be obtained. After combining the operations and using the 1 × 1 convolutional transform function $F_{1}$ to transform them:

(3) $$f=\delta (F_{1}([z^{h},z^{w}]))$$

In Equation (3), $z^{h}$ is the output of all channels with height *h*; $z^{w}$ is the output of all channels with width *w*; $\left[z^{h},z^{w}\right]$ is the continued combination operation along the spatial dimension; *δ* is the nonlinear activation function; and *f* is the intermediate feature mapping, which encodes the spatial information in the horizontal and vertical directions. After decomposing into $f^{h}$ and $f^{w}$ along the horizontal and vertical dimensions and using the 1 × 1 convolutional transform functions $F_{h}$ and $F_{w}$ to transform $f^{h}$ and $f^{w}$ into tensors with the same number of channels, respectively, the calculation process is:

(4) $$g^{h}=\sigma (Fh(f^{h}))\;;\;g^{w}=\sigma (Fw(f^{w}))$$

In Equation (4), *σ* is the sigmoid activation function. After expanding the output $g^{h}$ and $g^{w}$ as the dimensional weights of vertical and horizontal attention, the output feature image is:

(5) $$y_{{}_{c}}(i,j)=x_{c}(i,j)\times g_{c}^{h}(i)\times g_{c}^{w}(j)$$

In Equation (5), $g_{c}^{h}$ and $g_{c}^{w}$ are expanded by $g^{h}$ and $g^{w}$ as the dimensional weights of vertical and horizontal attention; $x_{c}$ is the feature map of the *c*th channel; and $y_{c}(i,j)$ is the output attention-weighted image.

### 2.5. Feature Fusion

To improve the feature extraction ability of the network and to enrich the feature expression, this study fused ResNet and Xception. Firstly, the outputs of ResNet and Xception networks were mixed pooling compared with the traditional pooling; mixed pooling introduces pooling selection coefficients during the training process, which in turn, determines the pooling method and changes the rules of pooling adjustment randomly. This method is better than the traditional single pooling method and is also beneficial in the prevention of overfitting to a certain extent. Then the features after hybrid pooling are combined along the spatial dimension, and finally, the fused features are pooled global averages to complete feature fusion. The feature fusion implementation process is shown in Equations (6) and (7).

(6) $$y_{out}^{p}=GAP\left(y^{res\_mix}⊕y^{x\_mix}\right)$$

(7) $$y_{kij}^{mix}=\lambda \cdot \underset{\left(p,q\right)\in R_{ij}}{\max}x_{kpq}+\left(1-\lambda \right)\cdot \frac{1}{\left|R_{ij}\right|}\sum_{\left(p,q\right)\in R_{ij}}x_{kpq}$$

In Equation (6), $y_{out}^{p}$ is the output feature map; *GAP* is the global average pooling; $y^{res\_mix}$ is the output of the ResNet network after hybrid pooling, which indicates the output of the Xception network after hybrid pooling; and $\left(y^{res\_mix}⊕y^{x\_mix}\right)$ indicates the combination operation of $y^{res\_mix}$ and $y^{x\_mix}$ along the spatial dimension.

In Equation (7), $y_{kij}^{mix}$ denotes the mixed pooling output value of the rectangular region, $R_{ij}$, associated with the kth feature map; *λ* is a random value of 0 or 1, indicating the choice of using maximum pooling or average pooling; $x_{kpq}$ denotes the element located at (p,q) in the rectangular region, $R_{ij}$, in the kth feature map; and $\left|R_{ij}\right|$ denotes the number of elements in the rectangular region, $R_{ij}$.

## 3. Dataset Introduction

**Figure 7:**
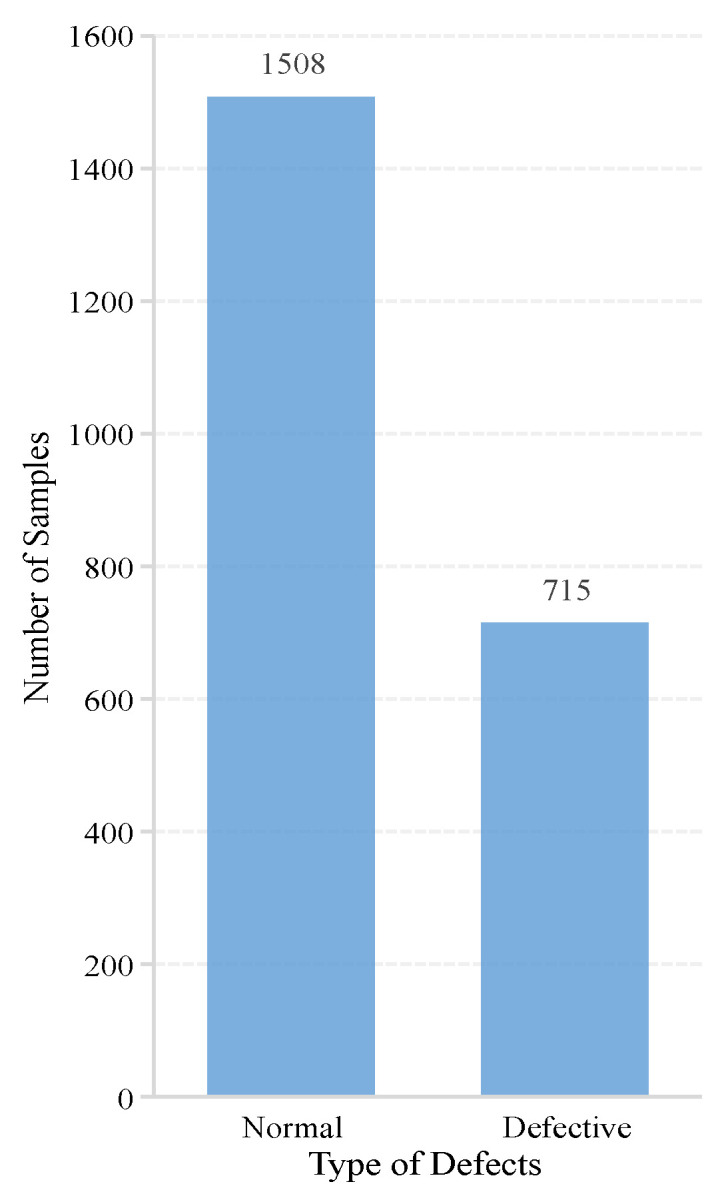
Distribution of defect types in dataset 1.

In this study, the two global public datasets of EL images used for the experiments were from the field of photovoltaic power generation. Dataset 1 [34] was a sample of 2624 PV cell images obtained from 44 PV modules with different degrees of defects, of which 18 modules were monocrystalline and 26 were polycrystalline; in addition, all samples were normalized by normalizing the size and view angle to 8-bit grayscale images of 300 × 300 pixels [35]. The critical detail of whether a PV cell is defective or not exhibited uncertainty due to the possible noise and unknown defect type of PV cells. Therefore, the image samples in the dataset were expertly labeled as “0%”, “33%”, “67%”, and “100%”, as four probabilities of the occurrence of PV cell defects. In order to check the performance of the model in identifying the presence of defects on the PV cell surface, we only selected two types of data, “0%” (without defects) and “100%” (with defects), to complete the experiment. Figure 7 shows the number of samples of these two types.

**Figure 8:**
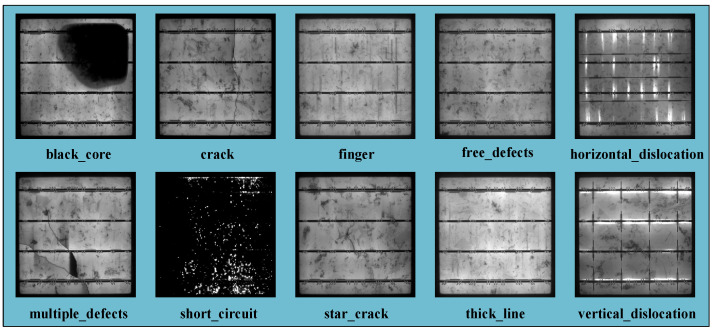
Diagram of different defect types.

Dataset 2 [36] was a high-resolution image sample of PV cell defects collected from actual industrial manufacturing, which was jointly published publicly by the Hebei University of Technology and Beijing University of Aeronautics and Astronautics. Compared with dataset 1, this dataset had higher resolution and more diverse and comprehensive types of anomalies, such as different types of defects including cracks (linear and stellate), broken grids, black cores, unaligned, thick lines, etc. Figure 8 shows the defects in dataset 2. In addition, any distortion issues in the images have been addressed, which helped to fully validate the model proposed in this paper.

**Figure 9:**
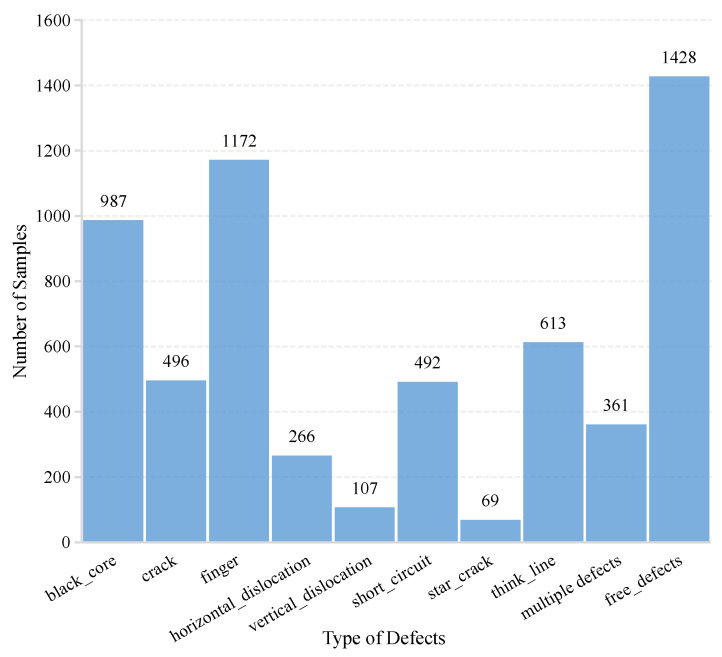
Distribution of defect types in dataset 2.

Therefore, based on the detection of the presence or absence of defects by the model, we further validated the performance of the model in identifying different types of defects on the PV cell surface on dataset 2. Figure 9 shows the number of samples with “no defects”, and the nine defect types in dataset 2.

According to the distribution of category samples in dataset 1 and dataset 2, two problems can be found: (1) the number of samples in different categories is unbalanced, with large disparities; (2) the number of images trained based on deep learning is relatively small. Therefore, in order to balance the distribution of samples within different categories and to make full use of the value of limited data, we adopted data enhancement and category weighting strategies.

### 3.1. Data Enhancement

Large-scale datasets are an important prerequisite for the successful application of deep learning techniques. Therefore, in this study, a series of stochastic changes was applied to the training set using a data enhancement strategy to improve the generalization ability of the employed deep learning model and to avoid overfitting problems. First, all images were normalized in order to improve the speed and possibly the accuracy of gradient descent for optimal solutions. Second, to obtain more images with different defect patterns, all the images were row–row swapped, randomly flipped vertically and flipped horizontally. We did not use random cropping and panning because some key regions that affect the model judgment would be cropped or panned, resulting in the model’s failure to learn the features during training. Furthermore, the blurring process reduced the influence of dark areas in EL images, and we randomly chose from Gaussian blur, motion blur and center blur. Finally, image brightness, contrast and saturation enhancement techniques were applied to the original EL images to produce new images with different and useful information.

### 3.2. Category Weights

As mentioned in the previous section, the dataset in this paper had the problem of data imbalance, and to address this problem, this study used type weights to weight the categories; the weight balance formula is shown in Section 3.1. Each sample was given a different weight value according to the number of samples in its category, and the category with a small number of samples is weighted more, i.e., the loss of sample misclassification is greater, which acts on the loss function during the training process, thus making the model pay more attention to the category with a small number of samples.

(8) $$w_{j}=\frac{n\_samples}{\left(n\_classes*n\_samples_{j}\right)}$$

where $w_{j}$ is the weight of category j; n_samples is the total number of samples in the dataset; n_classes is the number of categories; and $n\_samples_{j}$ is the total number of samples in category j.

## 4. Experimental Setting

For dataset partitioning, we selected 80% of the EL images as the training set and the remaining 20% as the test set; in addition, stratified sampling was used to randomly partition the original data while preserving the distribution of samples within different categories in the training and test sets. Table 1 shows the partitioning results of dataset 1, separated by the type of PV cells, and Table 2 shows the partitioning results of dataset 2.

The experiments were conducted with Ubuntu OS, an Intel i5-6600 processor, 16 GB RAM, and Nvidia RTX2080 latform for GPU, and the program was written in Python language based on the Tensorflow environment. The optimizer was Adam; the experimental batch size was set to 16, which means that the number of samples input to the model was 16 for each iteration; the learning rate was initialized to 0.01; the minimum learning rate was 0.0001; the maximum number of iterations was 2000; l2 regularization was introduced to alleviate overfitting; and the learning rate update strategy was the warm-up phase (warmp-up) using one-dimensional linear interpolation. The cosine annealing algorithm [36] was used after the warm-up phase, and the pseudo-code for the learning rate update is shown in Algorithm 1.
**Algorithm 1** Learning Rate Update Algorithm**1.** **Input:** Learning rate lr; minimum learning rate min_lr; initial learning rate init_lr; maximum number of iterations τ; warm-up iterations τ ***2.** β = 1**3.** while Termination conditions are not met, do**4.** if β < τ ***5.**  One-dimensional linear interpolation g = [0,τ *]**6.**  Update lr = g**7.**  β = τ ***8.**  else**9.**  Update lr = min_lr + (init_lr-min_lr)*((1 + cos($\pi \frac{\beta }{\tau }$))/2)**10.**  end if**11.**  β = β + 1**12.**  end while**13.**  **Output:** Update learning rate lr *

## 5. Results

### 5.1. Evaluation Metrics

Accuracy, recall, precision and *F*1 score are the four metrics commonly used to evaluate and compare good models; we chose to measure the classification effect and performance of our proposed model. Notably, for the accuracy, precision, recall and *F*1 scores, a larger value represents a better result.

Accuracy is an evaluation metric that approximates a model’s performance across all classes. It is calculated using the following formula:

(9) $$Accuracy=\frac{TP+TN}{TP+TN+FP+FN}$$

where *TP* and *TN* are the number of true positives and true negatives, respectively, and *FP* and *FN* are the number of false positives and false negatives, respectively.

The recall metric is the ratio between the correctly classified positives (true positive) and total number of positives (true positive and false negative). It is calculated using the following formula:

(10) $$Recall=\frac{TP}{TP+FN}$$

Precision is a metric which is calculated as the ratio between true positives and the total number of positives (true positives and false positives). It is calculated using the following formula:

(11) $$Precision=\frac{TP}{TP+FP}$$

The *F*1 score is a comprehensive evaluation metric which takes the harmonic mean of recall and precision. It is calculated using the following formula:

(12) $$F1\;score=2\times \frac{precision\times Recall}{precision+Recall}$$

### 5.2. Binary Classification Experiments

The surface of the normal PV cell EL images was uniform, although there were shadow areas or impurities in the background of the images and there were clear textured backgrounds, which were normal and could not be classified as having defective types, which puts some pressure on the model to identify defects. The defects on the surface of abnormal PV cells were different from the background in the image, but these defects were generally similar in appearance to the background in the EL image, so it was difficult to distinguish them.

In this subsection, performance evaluation experiments of the binary classification task using the final model are presented, including three comparative demonstrations to verify the effectiveness of the proposed model in the process of identifying the presence of defects in PV cells. First, the results of data enhancement are introduced to compare the performance of the model before and after the data enhancement strategy. Second, the proposed binary classification model was tested and compared with other commonly used neural network models to analyze the advantages of the proposed model in terms of quantitative results and qualitative results. Finally, to highlight the power of the proposed hybrid model again, we compared the model with the advanced PV defect recognition models in recent years.

#### 5.2.1. Comparison with Data Augmentation

Training deep learning models on small datasets is a major challenge because too small a training set can risk overfitting the module, which is a problem that needs to be addressed. Applying our proposed data augmentation strategy to the original training set gave the neural network model more valuable information to help train a superior classifier. The data enhancement treatment improved the accuracy of the model based on reducing the risk of overfitting; this reduced the variance between images and helped the model learn more representative feature representations, thus significantly improving the recognition accuracy. Experiments were conducted using ResNet152, Xception and the model proposed in this paper, and the training set images were enhanced by interchanging and flipping the image ranks from the image space level and by randomly varying the image brightness, contrast and saturation from the pixel value level. From the results presented in Table 3 and Table 4, the data enhancement strategy had the highest accuracy, leading to the conclusion that the chosen data enhancement operation is effective in improving the model performance.

#### 5.2.2. Comparison with Other Methods

To demonstrate the performance of our proposed model, we compared our model with the following methods for PV cell defect detection: (1) CNN, (2) VGG16, (3) MobileNetV2, (4) InceptionV3, (5) DenseNet121 and (6) InceptionResNetV2.

##### Quantitative Comparison

The quantitative results are shown in Table 5. In the experiments, our model was compared on the image classification evaluation metrics accuracy, *F*1 score, recall and precision. Notably, our model was based on ResNet152 and Xception, and the results show that it effectively utilizes the advantages of both to extract more representative features from images. Compared with six typical classification models, our model shows significant improvements in all four evaluation metrics. Notably, for a fair comparison, all these methods were retrained using the same settings as the proposed method.

##### Qualitative Comparison

**Figure 10:**
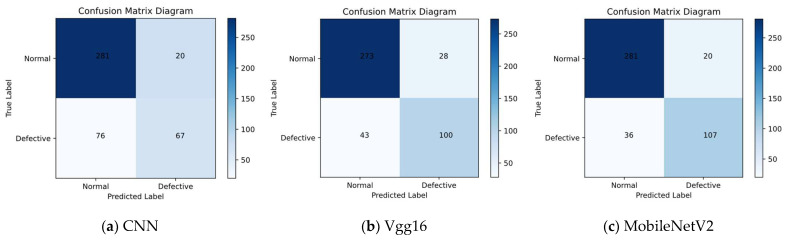

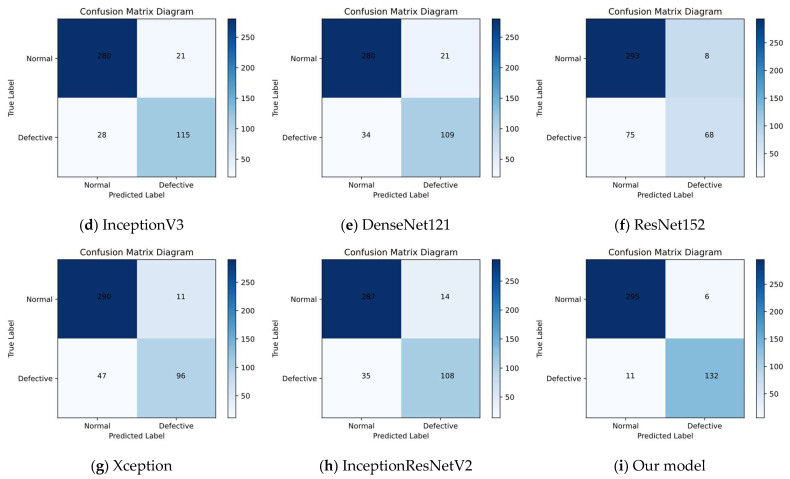
Confusion matrix diagrams for different models. (**a**) CNN (**b**) Vgg16 (**c**) MobileNetV2 (**d**) InceptionV3 (**e**) DenseNet121 (**f**) ResNet152 (**g**) Xception (**h**) InceptionResNetV2 (**i**) Our model.

The qualitative results are shown in Figure 10. To further validate that our model is more focused on the category imbalance problem, the confusion matrix was used to compare our model with other classification models more intuitively. In the test dataset, the distributions of both defect-free and defective types of data were unbalanced, and the amount of defect-free data was twice as large as that of defective data, thus requiring a classification model with better performance to make accurate judgments. Both CNN and ResNet152 models performed poorly on the entire test dataset, and the number of incorrect classifications was greater than the number of correct classifications. Although the other six models had improved various evaluation metrics compared with the first two models, they still performed poorly on the “defective” type, which exhibited a relatively small percentage of data. Figure 10i shows the classification performance of our model in a confusion matrix. The classification accuracy of our model in the “flawed” category was significantly improved, and the confusion matrix comparison results were sufficient to demonstrate a significant improvement in the accuracy of our model compared with the traditional classification model.

#### 5.2.3. Comparison with State-of-the-Art Methods

Our model was compared with some of the more effective methods for PV defect identification in recent years. For a fair comparison, these methods were chosen for experimentation and evaluation on the same dataset as our proposed model; the results of the method comparison are shown in Table 6.

(1) SVM [3]: the SVM was trained based on various features extracted from the EL images of solar cells; (2) L-CNN: a lightweight CNN architecture containing three convolutional layers, three pooling layers and a batch normalization layer added after the third pooling; (3) Light CNN: a novel convolutional neural network architecture which used light to automatically detect PV cell defects in electroluminescent images; (4) DFB-SVM: a CNN which extracted feature vectors of images and classified the extracted features with various combinations of connections using different machine learning methods; (5) Hessian matrix: a defect feature extraction method based on the Hessian matrix and a defect feature enhancement method based on a multi-scale line detector were used to improve the performance.

### 5.3. Multi-Classification Experiments

This subsection presents experiments on the performance evaluation of the multi-classification task using the final model, including three comparative demonstrations to verify the effectiveness of the proposed model in the process of identifying different defect types in PV cells. First, the proposed multi-classification model was tested and compared with other commonly used neural network models in terms of four common classification evaluation metrics; in addition, we demonstrated the performance effectiveness of all models on each type of data in detail. Second, to ensure the authenticity of the experimental process and the stability of the models, we demonstrate the changes in each model during the training process in the form of graphs. Finally, we conducted ablation experiments on the proposed models to again demonstrate the effectiveness of the models in the multi-classification task.

#### 5.3.1. Comparison with Other Methods

There has been considerable research carried out on the surface defects of PV cells in recent years, but most of the data used in these experiments only cover four or five types of defects; thus, it was particularly important to apply public datasets covering more different defect types in our study. The more types of defects, the more pressure on the model’s ability to identify them. In addition, the data of particularly subtle defect types, such as “think_line” and “crack”, pose a significant challenge to the model.

We compared the final model with the eight commonly used neural network models in terms of both comprehensive and detailed evaluation. Table 7 clearly shows that our proposed model had a significant improvement effect on the four classification evaluation indexes compared with other models, and the recognition accuracy reached 92.13%. These traditional models performed poorly on the multi-classification task in terms of classification ability and did not effectively address the problems brought by the solution dataset to meet the needs in practical application scenarios.

In order to be able to more clearly observe the details of the significant improvements in our proposed models, Table 8 details the recognition accuracy results of all models for each defect type; notably, the training parameters and datasets are identical for all models, and the bolded values in each column of the table mean the best results for that type.

The images exhibiting “black_core”, “horizontal_dislocation” and “short_circuit”, three different defect types in the images, had good discrimination, and all the models could accurately extract the feature representations of them in training, achieving good recognition results. The images exhibiting “finger”, “vertical_dislocation” and “free_defects” were not as clear; however, our proposed model still performed better, having more power to extract information from the image, which was the desired result. The other four types of defects were not as conducive: the defects were more similar to the background texture, and the “think_line” defects were particularly subtle and challenging to identify with the naked eye. However, our model had the advantage of its feature fusion, and the powerful feature representation extraction capability significantly improved the classification performance.

#### 5.3.2. Training Process

**Figure 11:**
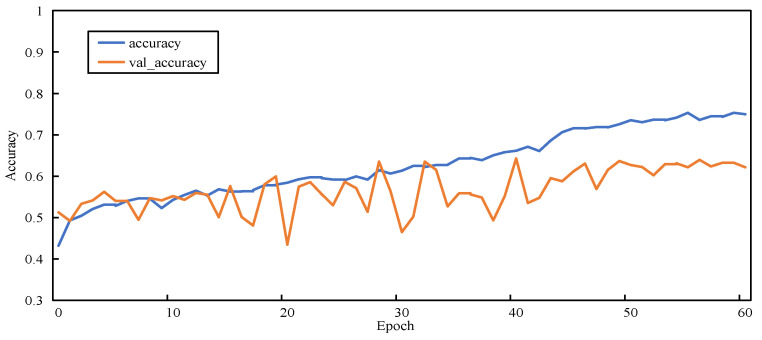
Accuracy variation graph of the CNN model.

**Figure 12:**
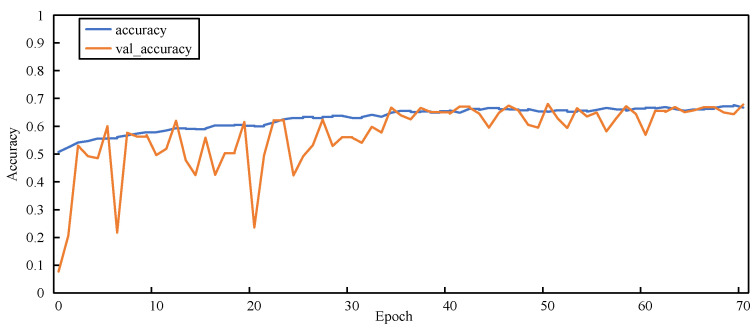
Accuracy variation graph of the ResNet152 model.

**Figure 13:**
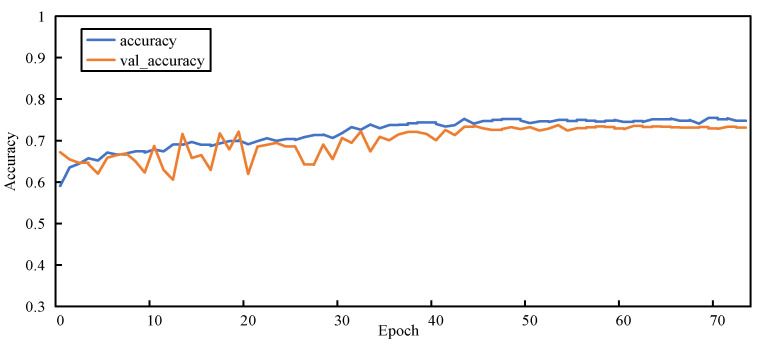
Accuracy variation graph of the Xception model.

**Figure 14:**
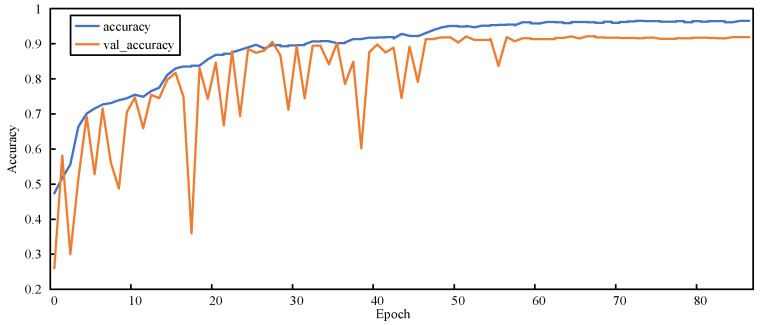
Accuracy variation graph of our model.

Figure 11, Figure 12, Figure 13 and Figure 14 show the changes in two metrics, accuracy and val_accuracy, of CNN, ResNet152, Xception, and the final model during the training process, respectively. Although the CNN and ResNet152 models eventually converged to a certain value, there were fluctuations throughout the whole process. Even the Xception model started to converge after 40 rounds of training and performed more stably, but the final convergence did not reach the ideal state. Our proposed model exhibited a large fluctuation in the early training period, and the model automatically adjusted the parameters, started to converge after 56 rounds of training, and was more stable in the late training period, with a significant improvement in the convergence value compared with other models.

#### 5.3.3. Ablation Study

To further verify the effectiveness of the model proposed in this paper, we performed ablation experiments to analyze the performance of our model on various metrics. The results are shown in Table 9. We fused the features of two models, ResNet152 and Xception, and added CA to significantly improve the parameters of the model in all aspects of metrics. For the problem of scant and unbalanced data, using a data enhancement strategy and class_weight approach to balance the weight of dataset types, our model greatly alleviated the problem of small samples of a certain class affecting the performance of the model and effectively solves the problem of unbalanced data classification, which is sufficient to prove that our proposed model to identify PV cell defects is effective.

## 6. Conclusions

In this paper, we have proposed a framework for the automatic detection of defective PV modules in EL images with a limited sample size based on deep learning. First, an effective data enhancement and category weight assignment method is proposed, which can generate a large number of high-resolution EL images for model training and solve the problem of uncategorized samples in the dataset. Then a hybrid neural-network-based defect detection model is proposed, which also combines the advantages of ResNet152 and Xception networks, and uses a feature fusion algorithm to extract more effective features of the images and incorporates attention to enhance the detection capability of the model. The experimental results demonstrate that our proposed model completed binary classification experiments and multi-classification experiments on two global public PV defect datasets, with significant improvements in several evaluation metrics compared with eight common single models. The numerical experimental results demonstrate the effectiveness of the model.

In the future, there are some directions in the EL field that deserve further research and development. First, more defective EL images need to be considered to increase the generalization capability of the model to assess the healthy operating state of PV cells. Then the rapid development of EL sensors and high-performance computing hardware can be used to deploy the models in practical large-scale PV plant application scenarios.

## 7. Data Availability

The following information was supplied regarding data availability:

Dataset 1 is available at GitHub: https://github.com/zae-bayern/elpv-dataset (accessed on 21 August 2022).

Dataset 2 is available at http://aihebut.com/col.jsp?id=118 (accessed on 15 June 2022).

The code is available at GitHub:

https://github.com/Zayn-Wang/Photovoltaic-cell-surface-defect-detection (accessed on 20 December 2022).

## Figures and Tables

**Table 1:** The numbers of dataset 1 given for the 80%/20% training/test split.

Photovoltaic Cell Type	Train		Test		Total
	0%	100%	0%	100%	
Monocrystalline	470	251	118	62	901
Polycrystalline	737	321	183	81	1322
Total	1207	572	301	143	2223

**Table 2:** The numbers of dataset 2 given for the 80%/20% training/test split.

Defect Category	Train	Test	Total
black_core	790	197	987
Crack	397	99	496
Finger	938	234	1172
horizontal_dislocation	213	53	266
vertical_dislocation	86	21	107
short_circuit	394	98	492
star_crack	56	13	69
think_line	491	122	613
multiple defects	289	72	361
free_defects	1143	285	1428
Total	4797	1194	5991

**Table 3:** Results before using the enhancement procedure.

Model	Accuracy	*F*1 Score	Recall	Precision
ResNet152	0.7973	0.7313	0.7110	0.8089
Xception	0.8288	0.7914	0.7765	0.8182
Our model	0.9414	0.9317	0.9238	0.9410

**Table 4:** Results after using the enhancement procedure.

Model	Accuracy	*F*1 Score	Recall	Precision
ResNet152	0.8221	0.7702	0.7476	0.8346
Xception	0.8694	0.8385	0.8174	0.8789
Our model	0.9617	0.9557	0.9516	0.9603

**Table 5:** Comparison with other methods.

Model	Accuracy	*F*1 Score	Recall	Precision
CNN	0.7838	0.7184	0.7010	0.7786
Vgg16	0.8401	0.8115	0.8031	0.8226
MobileNetV2	0.8739	0.8510	0.8409	0.8645
InceptionV3	0.8896	0.8720	0.8672	0.8773
DenseNet121	0.8761	0.8546	0.8462	0.8651
InceptionResNetV2	0.8896	0.8682	0.8544	0.8883
Our model	0.9617	0.9557	0.9516	0.9603

**Table 6:** Comparison with state-of-the-art methods.

Reference	Model	Accuracy
[35]	SVM	82.44%
[37]	L-CNN	89.33%
[25]	Light CNN	93.02%
[37]	DFB-SVM	94.52%
[24]	Hessian matrix	93.00%
	Our model	96.17%

**Table 7:** Comparison with common methods.

Model	Accuracy	*F*1 Score	Recall	Precision
DenseNet121	0.7655	0.7244	0.7108	0.7482
MobileNetV2	0.7680	0.7298	0.7186	0.7479
Xception	0.7379	0.6792	0.6678	0.7077
ResNet152	0.6809	0.5837	0.5933	0.6153
Vgg16	0.7772	0.7326	0.7181	0.7751
InceptionV3	0.7605	0.7113	0.6945	0.7540
CNN	0.6432	0.5737	0.5824	0.6111
InceptionResNetV2	0.7898	0.7662	0.7551	0.7819
Our model	0.9213	0.8898	0.8961	0.8872

**Table 8:** Recognition accuracy of different models for each defect type.

Model	D1	D2	D3	D4	D5	D6	D7	D8	D9	D10
DenseNet121	0.9848	0.5152	0.8205	1.0000	0.9048	1.0000	0.3077	0.4590	0.3333	0.7825
MobileNetV2	0.9898	0.4949	0.7778	1.0000	0.9524	1.0000	0.3077	0.4426	0.4028	0.8175
Xception	0.9949	0.4949	0.7393	1.0000	0.9048	1.0000	0.1538	0.4098	0.1806	0.8000
ResNet152	0.9898	0.4141	0.7564	1.0000	0.9048	1.0000	0	0.1066	0	0.7614
Vgg16	0.9797	0.6768	0.7991	1.0000	0.9048	1.0000	0.2308	0.3279	0.4306	0.8316
InceptionV3	0.9949	0.5354	0.7778	1.0000	0.9048	0.9898	0.2308	0.4262	0.2639	0.8211
CNN	0.9645	0.4343	0.7521	1.0000	0.9048	0.9898	0.0769	0.0492	0.0139	0.6386
InceptionResNetV2	0.9898	0.5253	0.7778	1.0000	0.9048	0.9898	0.5382	0.5000	0.4722	0.8526
Our model	1.0000	0.8081	0.9188	1.0000	0.9524	1.0000	0.8462	0.9016	0.5694	0.9649

Note: D1–D10 represent 10 types of defects: black_core, crack, finger, horizontal_dislocation, vertical_dislocation, short_circuit, star_crack, think_line, multiple defects and free_defects, respectively.

**Table 9:** Results of the ablation experiments.

Model	Accuracy	*F*1 Score	Recall	Precision
ResNet152	0.6809	0.5837	0.5933	0.6153
Xception	0.7379	0.6792	0.6678	0.7077
ResNet152 + Xception	0.8702	0.8689	0.8624	0.8349
ResNet152 + Xception + class_weight	0.8875	0.8625	0.8478	0.8598
ResNet152 + Xception + class_weight + Data Enhancement	0.9032	0.9143	08970	0.8744
Our model	0.9213	0.8898	0.8961	0.8872

## Data Availability

Not applicable.
